# Major postoperative complications are associated with impaired long-term survival after gastro-esophageal and pancreatic cancer surgery: a complete national cohort study

**DOI:** 10.1186/s12893-016-0149-y

**Published:** 2016-05-18

**Authors:** Eirik Kjus Aahlin, Frank Olsen, Bård Uleberg, Bjarne K. Jacobsen, Kristoffer Lassen

**Affiliations:** Department of GI and HPB surgery, University Hospital of Northern Norway, 9038 Breivika, Tromsø, Norway; Department of Clinical Medicine, University of Tromsø - The Arctic University of Norway, Tromsø, Norway; Centre of Clinical Documentation and Evaluation, Northern Norway Regional Health Authority, Tromsø, Norway; Department of Community Medicine, University of Tromsø - The Arctic University of Norway, Tromsø, Norway

**Keywords:** Postoperative complications, Surgery, Neoplasms

## Abstract

**Background:**

Some studies have reported an association between complications and impaired long-term survival after cancer surgery. We aimed to investigate how major complications are associated with overall survival after gastro-esophageal and pancreatic cancer surgery in a complete national cohort.

**Methods:**

All esophageal-, gastric- and pancreatic resections performed for cancer in Norway between January 1, 2008, and December 1, 2013 were identified in the Norwegian Patient Registry together with data concerning major postoperative complications and survival.

**Results:**

When emergency cases were excluded, there were 1965 esophageal-, gastric- or pancreatic resections performed for cancer in Norway between 1 January 2008, and 1 December 2013. A total of 248 patients (12.6 %) suffered major postoperative complications. Complications were associated both with increased early (90 days) mortality (OR = 4.25, 95 % CI = 2.78–6.50), and reduced overall survival when patients suffering early mortality were excluded (HR = 1.23, 95 % CI = 1.01–1.50).

**Conclusions:**

Major postoperative complications are associated with impaired long-term survival after gastro-esophageal and pancreatic cancer surgery.

## Background

Major complications after surgery have negative effects on health-related quality of life [1], length-of-stay [2] and resource utilization [2]. Major complications may preclude or delay adjuvant cancer treatment [3]. In addition, long-term survival may be negatively affected [4–7].

Khuri and co-workers [5] found that patients experiencing complications from surgery had a markedly reduced long-term survival even when those who died within 30 days after surgery were excluded from analysis. As summarized in a recent meta-analysis, these findings have been corroborated by several reports, but others again have failed to show this connection [6]. It has been suggested that major complications could have long-standing suppressive effects on a patient’s immune system and thereby rendering them more susceptible to cancer recurrence [4, 5, 7–9].

Investigating a possible long-standing detrimental effect of postoperative complications on survival after cancer surgery is challenging: Major complications after modern surgery are relatively uncommon and therefore large cohorts are needed for analysis. There is no consensus on the correct cut-off for early mortality and there may be several factors that affect both susceptibility to complications and decreased overall survival - factors that have to be adequately adjusted for. Naturally, an interventional trial is impossible to conduct.

Patient’s level of education is a known indicator of important factors like physical activity and smoking habits [10], factors that are associated with both increased morbidity and impaired survival after surgery [11].

The aim of this study was to investigate whether major complications after gastro-esophageal and pancreatic surgery for cancer are associated with impaired long-term survival or early mortality only. We also aimed to investigate if such an association was influenced by patient’s level of education.

## Methods

### Cohort identification

A database of surgical procedures, major postoperative complications and survival was extracted from the Norwegian Patient Registry (NPR). The Norwegian Patient Registry enables patients to be tracked from one stay to another, thus allowing for identification of an early complication occurring at a local hospital following transfer from a tertiary hospital where index surgery had been performed. All Norwegian hospitals must submit data to the Norwegian Patient Registry for registry and reimbursement purposes. In the present study, we included data from admissions during 2008–2013. Data on educational level were extracted from Statistics Norway, the Norwegian central bureau of statistics.

We identified all resections of the esophagus, stomach and pancreas in the six-year period from January 1, 2008 to December 1, 2013. Complications within 28 days after surgery (to the end of 2013) are therefore included in the data material. Data on overall survival until June 30, 2014 were retrieved, thus even the last patient subject to surgery included would have seven months follow-up, if not dying. Operations and reoperations were identified from their Nomesco classification of Surgical Procedures (NCSP) code (2014). NCSP is available for download at http://www.nordclass.se/ncsp.htm.

Only cases with an appropriate cancer diagnosis (ICD-10: C15*, C16* and C25* respectively, where * denotes all sub-codes) applied within eight weeks from surgery were included. Emergency cases were excluded to obtain a cohort of patients that were reasonably fit at index surgery.

Overall survival was defined as survival from index surgery. Major complications were defined as equivalent to Accordion score IV or higher, equaling Clavien-Dindo score IIIb or higher [12, 13], i.e. a re-operation in general anesthesia for a complication, and/or single- or multiple organ failure [12]. We did not attempt to identify less severe complications, as these would be difficult to extract from Norwegian Patient Registry data.

All stays in this database (containing an eligible index operation) were coupled with any subsequent stay at any Norwegian hospital with an admission date within 28 days from discharge from the index stay. Hospital stays (single or coupled) containing one or more major complications were identified. This was done by identification of one of the following procedure codes at index or subsequent stay (NCSP): Reoperation for wound dehiscence (JWA00), for deep infection (JWC00/01), for deep hemorrhage (JWE00/01/02), for anastomotic dehiscence (JWF00/01), reoperation for other causes (JWW96/97/98) or if a tracheostomy was performed (GBB00/03). Also, a major complication was identified from the use of one of the following diagnoses during index or subsequent stays (ICD-10): Bleeding, hematoma or circulatory chock following a procedure (ICD-10: T81.0, T81.1, T81.3).

### Validation of algorithm

The algorithms to identify complications were constructed in several wider variations and then validated against hand searched patient files. A tendency to over-score complications (identifying Accordion III or Clavien-Dindo IIIa as “major complications”) when using the entire complications section (ICD-10: T81*) was avoided when some sub-codes (ICD-10: T81.2, T81.4, T81.5, T81.6, T81.7, T81.8 and T81.9) were removed from the algorithm. A comparison of the yield from our refined search strings with hand searched patient files in a four-year cohort at our own hospital (150 patients) showed a 100% match in both number of resections and rate of major complications (Accordion IV or higher).

### Definition of variables

Age at index surgery was analyzed both as a continuous variable and with a cut-off of 65 years. Educational level was divided into higher and lower education. Higher education was defined as education beyond primary and lower secondary school. Surgical resections were stratified into esophageal-, gastric- and pancreatic resections.

### Statistics

**Fig. 1:**
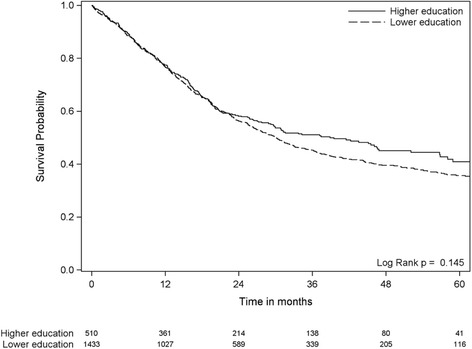
The association between educational level and overall survival. Kaplan-Meier plots with subjects at risk. Survival probability on the Y-axis and time in months on the X-axis. Dotted lines denotes patients with lower education. Solid lines denotes patients with higher education

**Fig. 2:**
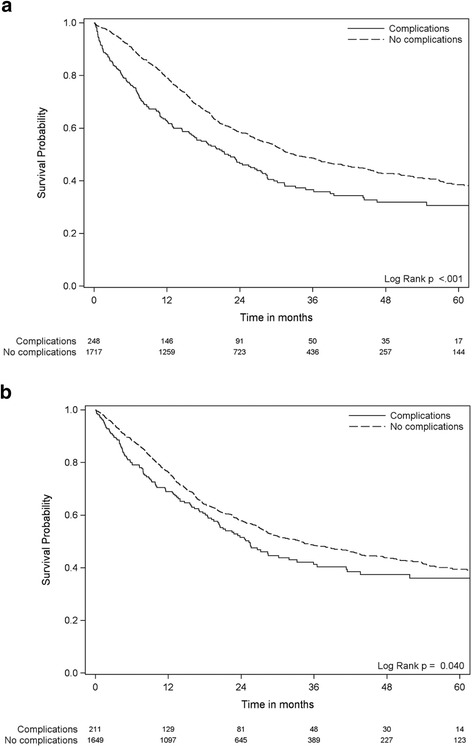
The association between major postoperative complications and overall survival. Kaplan-Meier plots with subjects at risk. Survival probability on the Y-axis and time in months on the X-axis. Dotted lines denotes patients without any postoperative complications. Solid lines denotes patients who suffered one or more major postoperative complication. **a** All patients included. **b** Patients alive more than 90 days only

Statistical analyses were performed with SAS statistics software, SAS Institute, Cary, NC, USA. For comparison of characteristics between different groups and categories, Student t-test for continuous data and Pearson chi-square test for categorical data were used. Logistic regression was used for analyzing associations between age, gender, educational level, type of resection and the risk of a major postoperative complication as well as early (90 days) mortality. Kaplan-Meier survival plots with log-rank test and Cox proportional hazard (PH) regression analysis were used for analysis of overall survival. Both methods were used, as the proportional hazards assumption for Cox regression (constant relative hazards over time) was not met when analyzing the relationships between educational level and survival (Fig. 1) and when analyzing the relationship between complications and survival (Fig. 2). Cox proportional hazard regression analysis using attained age as the time variable was also used as a supplement, as the proportional hazards assumption was met in this situation. *P*-values according to the log-rank-test are given in the figures (the Kaplan-Meier survival plots) whereas p-value according to the Cox regression analyses (including the analyses adjusted for possible confounders) in Table 3. *P*-values <0.05 were considered statistically significant.

### Ethics

Centre of Clinical Documentation and Evaluation has concession from the Norwegian Data Protection Authority and confidentiality exemption from the Regional Committee for Medical and Health Research Ethics (REK –Northern chapter). The concession provides access to unique personal data from Norwegian Patient Registry with information about patients treated at Norwegian hospitals in the period 2008–2013. Encrypted patient serial numbers makes it possible to describe patient pathways involving several hospitals and over several years. The concession also includes permission to pair education data from Statistics Norway.

## Results

### Patient selection and characteristics

A total of 3080 esophageal-, gastric and pancreatic resections were performed between 1January 2008 and 1December 2013. Of these, 2792 resections were scheduled. Of the 2792 scheduled resections, 1965 were performed for cancer; 331 esophageal resections (16.8 %), 974 gastric resections (49.6 %) and 660 pancreatic resections (33.6 %). Seventy percent of the gastric resections and almost all of the esophageal (99 %) and pancreatic resections (98 %) were performed at the six university hospitals (only four university hospitals perform esophageal resections). Forty percent of esophageal and half of the pancreatic resections were performed at Norway’s largest hospital, Oslo University Hospital (OUS). Only 13% of the gastric resections were performed at OUS.

During the follow-up period, 975 (49.6 %) patients died. Median age at index operation was 68 years. A total of 248 patients (12.6 %) suffered one or more major postoperative complication and 37 (14.9 %) of these patients died within 90 days. Consequently, 1717 patients (87.4 %) did not suffer any major complications within 28 days after surgery and 68 (4.0 %) of these patients died within 90 days. Estimated median survival in the entire cohort was 31 months (Table 1) and the estimated five-year survival rate was 37.3 %.

**Table 1:** Cohort characteristics: Number of patients, age, gender, educational level, percentage with complications and estimated median survival according to type of resection

	Esophageal resections	Gastric resections	Pancreatic resections	All patients
Number of patients	331	974	660	1965
Age above 65 years (%)	46.8	67.7	56.4	60.4
Male gender (%)	77.6	62.4	52.7	61.7
Lower educational level (%)	69.5	78.5	68.9	73.7
Complication-rate (%)	17.2	11.1	12.6	12.6
90-day mortality (%)	5.7	6.1	3.9	5.3
Estimated median survival in months	46	36	23	31

### Major postoperative complications

There were no statistically significant linear changes in the rate of complications from 2008 to 2013 (*p* = 0.319). The highest (17.2 %) and lowest (11.1 %) complication rate was seen in esophageal resections and gastric resections, respectively (Table 2). Type of resection and lower educational level (OR = 1.41, 95 % CI = 1.02–1.95, *p* = 0.039) were associated with major postoperative complications. The association with educational level was also present when adjusted for age, gender and type of resection (OR = 1.53, 95 % CI = 1.10–2.13, *p* = 0.013).

**Table 2:** Age, gender, educational level and type of resection, and association with major postoperative complications

Variable	Categories	Percentage^a^	OR (95 % CI)^b^	*p*-value
Age	Above 65 years	12.2	0.91 (0.70–1.20)	0.515
	65 years or less	13.2	1.0	
Gender	Male	13.7	1.29 (0.98–1.72)	0.071
	Female	10.9	1.0	
Educational level	Lower education	13.5	1.41 (1.02–1.95)	0.039
	Higher education	10.0	1.0	
Type of resection	Esophageal	17.2	1.67 (1.18–2.36)	0.004
	Pancreatic	12.6	1.15 (0.85–1.56)	0.358
	Gastric	11.1	1.0	

^a^Percentage that suffered one or more major postoperative complications

^b^Odds ratio, with 95 % confidence interval, for suffering one or more major postoperative complications

### Early mortality

One-hundred-and-five (5.3 %) patients died within 90 days after surgery. Suffering one or more postoperative complications was strongly associated with 90-day mortality (OR = 4.25, 95 % CI = 2.78–6.50, *p* < 0.001).

### Overall survival

There was no association between educational level and overall survival (*p* = 0.145) (Fig. 1) and this conclusion did not change after multivariable adjustment for age, gender and type of resection (HR = 1.06, 95 % CI = 0.91–1.23, *p* = 0.475).

**Fig. 3:**
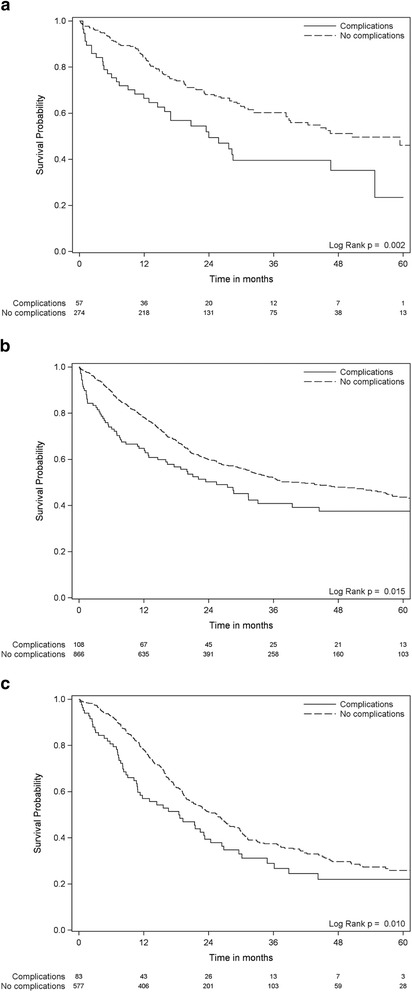
The association between major postoperative complications and overall survival. Esophageal, gastric and pancreatic resections analyzed separately. Kaplan-Meier plots with subjects at risk. Survival probability on the Y-axis and time in months on the X-axis. Dotted lines denotes patients without any postoperative complications. Solid lines denotes patients who suffered one or more major postoperative complication. **a** Esophageal resections. **b** Gastric resections. **c** Pancreatic resections

Postoperative complications were negatively associated with overall survival both with all patients included (Fig. 2, panel a) (*p* < 0.001) and when the first 90 days of the follow-up period (Fig. 2, panel b) (*p* = 0.040) were excluded. The survival curves (Fig. 2) suggest that the impact of major complications attenuate with time from the index surgery and p-value for interaction with time of follow up was <0.001. The estimated five-year survival rate was 38.2 % in patients who did not suffer any major postoperative complications compared to 30.6 % in patients who did. Major postoperative complications were associated with 23 % higher long term (i.e., excluding early) mortality (HR = 1.23, 95 % CI = 1.01–1.40, *p* = 0.040) (Table 3). Multivariable adjustment for age, gender, type of resection and educational level did not alter the association between major postoperative complications and overall survival (Table 3). Neither did use of attained age (instead of time in study) as the time variable in the Cox regression analysis. There were no significant interaction between type of resection and complications in the survival analysis, i.e., the association between complications and survival were not statistically significantly different according to type of resection. The association between postoperative complications and survival was similar across all types of resections (Fig. 3). Neither hospital volume (OUS vs. other university hospitals; analyzed for esophageal and pancreatic resections) or hospital teaching status (university hospital vs. non-university hospital; analyzed for gastric resections) did affect the association between complications and survival (data not shown).

**Table 3:** The association between postoperative complications and overall survival, with hazard ratio (HR) and 95 % confidence interval (95 % CI)

	Unadjusted			Multivariable adjusted for age, gender and type of resection			Multivariable adjusted for age, gender, resection and educational level		
	HR	95 % CI	*p*-value	HR	95 % CI	*p*-value	HR	95 % CI	*p*-value
All patients	1.48	1.24–1.76	<0.001	1.51	1.27–1.79	<0.001	1.50	1.26–1.79	<0.001
Only patients alive >90 days	1.23	1.01–1.50	0.040	1.26	1.03–1.53	0.023	1.26	1.03–1.54	0.022

## Discussion

We present a complete national six-year cohort of gastro-esophageal and pancreatic resections for cancer in Norway with major complications and survival. Suffering one or more major postoperative complications was associated with both considerably increased early mortality and statistically significant decreased long-term survival. Educational level did not affect the relationship between complications and survival.

Several studies have demonstrated an association between postoperative complications and decreased survival [5, 6]. This has led to theories suggesting an immune-suppressive effect of postoperative complications that might lead to cancer recurrence [5, 8, 9]. A recent meta-analysis reported a hazard ratio (HR) of 1.28 for decreased overall survival after any postoperative complication [6], the cut-offs for early mortality were not reported [6]. We found a similar risk of decreased survival associated with major complications if patients suffering early mortality were excluded (HR = 1.23). In this large, nationwide population-based cohort, we found some evidence to support theories concerning long-standing detrimental effects of complications on survival after gastro-esophageal and pancreatic cancer surgery.

Most studies exploring the issue of postoperative complications and long-term survival have either not used a cut-off at all or a 30-days mortality cut-off to exclude patients with fatal complications [5, 6, 8, 9]. Only a few studies report a cut-off for early mortality of 90-days [7]. Therefore, patients who died later than 30 days, but still arguably as a direct result of their complications may still have been included in the analysis, making it more difficult to address a possible long-standing detrimental effect of non-fatal complications.

Reductions in both overall survival and disease-free survival have been observed in colorectal cancer patients who suffered major complications [7, 8, 14]. In a large study of colon cancer patients, complications were associated with precluded or delayed adjuvant chemotherapy [3]. In the same study, complications were not associated with reduced survival if adjuvant chemotherapy were given within appropriate time-limits [3]. While chemotherapy is an important element to achieve increased survival in resectable stage III and IV colon cancer [15, 16], the effect of adjuvant chemotherapy on long-term survival in gastro-esophageal and pancreatic cancer is variable and less certain [17, 18].

The avoidance of postoperative complications is of utmost importance as complications adversely affect health-related quality of life [1] and survival [5, 6]. Complications may preclude or delay adjuvant chemotherapy [3]. Do complications make patients more susceptible to cancer recurrence and therefore cause decreased longevity? Large prospective registries with detailed information on disease-stage, comorbidity, time of recurrence and cause of death are needed to fully investigate this question. The registries used for our study contain complete national data and a large number of patients but lack information on cancer stage and disease-specific survival.

## Conclusions

In a national setting, major postoperative complications are associated with both early mortality and decreased long-term survival after gastro-esophageal and pancreatic cancer surgery. Systematic quality improvement to avoid complications may improve the poor prognosis associated these cancers.

### Ethics approval and consent to participate

Centre of Clinical Documentation and Evaluation has concession from the Norwegian Data Protection Authority and confidentiality exemption from the Regional Committee for Medical and Health Research Ethics (REK –Northern chapter). The concession provides access to unique personal data from Norwegian Patient Registry with information about patients treated at Norwegian hospitals in the period 2008–2013. Encrypted patient serial numbers makes it possible to describe patient pathways involving several hospitals and over several years. The concession also includes permission to pair education data from Statistics Norway.

### Availability of data and materials

Centre of Clinical Documentation and Evaluation has concessions that provide access to unique personal data from Norwegian Patient Registry. According to Norwegian rules and regulations, these data cannot be made publically available.

## Abbreviations

NPR: Norwegian Patient Registry
NCSP: Nomesco Classification of Surgical Procedures
ICD-10: International Classification of Diseases, 10^th^ edition
OUS: Oslo University Hospital
